# Dietary supplementation of exopolysaccharides from *Lactobacillus rhamnosus* GCC-3 improved the resistance of zebrafish against spring viremia of carp virus infection

**DOI:** 10.3389/fimmu.2022.968348

**Published:** 2022-08-05

**Authors:** Mingxu Xie, Yu Li, Rolf Erik Olsen, Einar Ringø, Yalin Yang, Zhen Zhang, Chao Ran, Zhigang Zhou

**Affiliations:** ^1^ Sino-Norway Joint Lab on Fish Gut Microbiota, Institute of Feed Research, Chinese Academy of Agricultural Sciences, Beijing, China; ^2^ Norway-China Joint Lab on Fish Gastrointestinal Microbiota, Institute of Biology, Norwegian University of Science and Technology, Trondheim, Norway; ^3^ Laboratory of Gene Therapy, Department of Biochemistry, College of Life Sciences, Shaanxi Normal University, Xi’an, China; ^4^ Norwegian College of Fisheries Science, Faculty of Biosciences, Fisheries and Economics, University of Tromsø (UiT) The Arctic University of Norway, Tromsø, Norway; ^5^ Key Laboratory for Feed Biotechnology of the Ministry of Agriculture and Rural Affairs, Institute of Feed Research, Chinese Academy of Agricultural Sciences, Beijing, China

**Keywords:** GCC-3 EPS, gut microbiota, spring viremia of carp virus, type I IFN, zebrafish

## Abstract

Spring viremia of carp virus (SVCV) can cause high mortality of fish. The aim of this study was to investigate the effects of *Lactobacillus rhamnosus* GCC-3 exopolysaccharides (GCC-3 EPS) on zebrafish (*Danio rerio*) infected with SVCV and elucidate the underlying mechanisms. Zebrafish were fed with a control diet or diet supplemented with 0.5% and 1% of GCC-3 EPS for 2 weeks. The results showed that supplementation of GCC-3 EPS significantly improved the survival rate of zebrafish compared with the control group. In addition, dietary 0.5% and 1% GCC-3 EPS significantly up-regulated the expression of genes related to type I interferon (IFN) antiviral immunity. Consistent with *in vivo* results, GCC-3 EPS significantly inhibited SVCV replication in zebrafish embryonic fibroblast (ZF4) cells while significantly increased the expression of type I IFN signaling pathway related genes. Furthermore, knocking down TANK-binding kinase 1 significantly blocked the antiviral effect of GCC-3 EPS. Dietary GCC-3 EPS improved gut microbiota, and the culture supernatant of GCC-3 EPS-associated microbiota significantly inhibited SVCV replication in ZF4 cells compared with the control-microbiota counterpart. In conclusion, our results indicate that dietary GCC-3 EPS can improve the resistance of zebrafish against SVCV infection, and the mechanism may involve enhanced type I interferon signaling.

## Introduction

In recent years, the aquaculture industry has been affected by the outbreak of viral diseases (1, 2). Spring viremia of carp is caused by spring viremia of carp virus (SVCV) of a single-stranded RNA virus belonging to a member of the genus *Sprivivirus* of the family Rhabdoviridae and results in high mortality of juvenile carps with no effective commercial therapy (3–5). Therefore, the World Organization for Animal Health identifies SVCV as the pathogen of infectious diseases that must be reported (2, 6). Interferons (IFN) are widely expressed cytokines with antiviral effects and are the first line of defense against viral infections (7). To date, large numbers of IFN have been discovered, which can be classified into three subgroups as type I IFN, type II IFN, and type III IFN (8). Type I IFN system plays an important role in the antiviral immune response of fish (9). Consistent with the function in mammals, type I IFN in fish can directly inhibit the replication of virus by inducing the expression of antiviral proteins (9–11).

Gut microbiota has been proved to interact with the host during virus infection (12, 13). Studies have indicated that the removal of gut microbiota increases viral infection in antibiotic-treated or germ-free mice, suggesting the importance of commensal microbiota in anti-viral immunity (13–15). Consistent with these findings in mammals, Galindo-Villegas et al. found that conventional zebrafish (*Danio rerio*) had higher resistance to spring viremia of carp virus (SVCV) infection than germ-free counterparts, supporting that the gut microbiota contributed to fish antiviral capabilities (16). A recent study reported that the predominant commensal bacterium *Cetobacterium somerae* improved the antiviral immunity of zebrafish after SVCV infection and the underlying mechanism may involve type I IFN signaling (4), suggesting the contribution of commensal bacterium to the antiviral effect of microbiota. Moreover, some studies show that probiotics can significantly improve the survival rate after the virus challenge (13). For example, *Bacillus subtilis* 7k has been shown to be able to inhibit iridovirus infection in grouper (*Epinephelus fuscoguttatus* × *E. lanceolatus*) (17). In addition, *Clostridium butyricum* can protect gibel carp (*Carassius auratus gibelio*) from *Carassius auratus* herpesvirus infection (18). *Lactobacillus* comprises an important group of probiotics for both humans and animals. The antiviral effect of *Lactobacillus* has been reported in mammals (19–22). Similarly, some studies showed that dietary *Lactobacillus* can protect fish against virus infection (23, 24). The exopolysaccharides (EPS) of *Lactobacillus* have been found to play an important role in the antiviral function in many studies in mammals (25–29). However, there is little information about the antiviral effect of *Lactobacillus* EPS in fish.

In this study, we investigated the effects of dietary supplementation of EPS from *Lactobacillus rhamnosus* GCC-3 on zebrafish against SVCV infection. In addition, GCC-3 EPS was added to ZF4 cells at different concentrations to evaluate its effect on SVCV replication *in vitro*, and the effect of GCC-3 EPS on the type I IFN signaling pathway was investigated. The gut microbiota of zebrafish fed with GCC-3 EPS was measured by 16S ribosomal RNA (16S *r*RNA) sequencing and the effect of gut microbiota-mediated antiviral effect was investigated. To our knowledge, this is the first study about the effects of *Lactobacillus* EPS on the antiviral immunity of fish. Our findings demonstrated that dietary GCC-3 EPS improved the resistance of zebrafish against SVCV infection, which involved enhanced anti-viral IFN immunity after viral infection.

## Materials and methods

### *Lactobacillus rhamnosus* GCC-3 exopolysaccharides (GCC-3 EPS)

*Lactobacillus rhamnosus* GCC-3 was purchased from the China Center of Industrial Culture Collection and preserved by the China-Norway Joint Lab of Fish Gastrointestinal Microbiota of the Chinese Academy of Agricultural Sciences. The culture of GCC-3 and extraction and purification of GCC-3 EPS were conducted according to previous protocols (26).

### Fish husbandry and feeding

Zebrafish were provided by China Zebrafish Resource Center. All zebrafish were housed in the laboratory for 2 weeks to reduce stress responses before the start of the experiment. All experimental protocols of zebrafish were agreed the Institute of Feed Research of the Chinese Academy of Agricultural Sciences chaired by the China Council for Animal Care (No. 2018-AF-FRI-CAAS-001). A total of 180 2-month-old zebrafish with the same initial weight were randomly allocated into three groups, each with three repeating tanks and 20 fish. The control diet and GCC-3 EPS diet (0.5% and 1% of GCC-3 EPS) were fed at 6% of body weight every day. The feed formula is shown in **Table 1**. Water quality was monitored every day with a temperature of 28.5 ± 0.5 °C, and a pH of 6.6 - 7.1. The dissolved oxygen was not less than 6.05 mg O_2_/L, and the nitrate level was less than 0.11 mg N/L.

**Table 1:** Ingredients and proximate compositions of the experimental diets (g/100 g dry diet).

Ingredient	Control	0.5% GCC-3 EPS	1% GCC-3 EPS
Casein	40.00	40.00	40.00
Gelatin	10.00	10.00	10.00
Dextrin	28.00	28.00	28.00
GCC-3 EPS	0.00	0.50	1.00
Soybean oil	6.00	6.00	6.00
Lysine	0.33	0.33	0.33
VC phosphate ester	0.10	0.10	0.10
Vitamin mix^a^	0.40	0.40	0.40
Mineral mix^b^	0.40	0.40	0.40
CaH_2_PO_4_	2.00	2.00	2.00
Choline chloride	0.20	0.20	0.20
Sodium alginate	2.00	2.00	2.00
Microcrystalline cellulose	4.00	4.00	4.00
Zeolite	6.57	6.08	5.58
Total	100	100	100
Crude protein	42.19	42.19	42.19
Crude lipid	6.09	6.09	6.09
Total energy (KJ/g)	18.55	18.55	18.55

a Containing the following (g/kg vitamin premix): thiamine, 0.438; riboflavin, 0.632; pyridoxine·HCl, 0.908; d-pantothenic acid, 1.724; nicotinic acid, 4.583; biotin, 0.211; folic acid, 0.549; vitamin B-12, 0.001; inositol, 21.053; menadione sodium bisulfite, 0.889; retinyl acetate, 0.677; cholecalciferol, 0.116; dl-α-tocopherol-acetate, 12.632.

b Containing the following (g/kg mineral premix): CoCl_2_·6H_2_O, 0.074; CuSO_4_·5H_2_O, 2.5; FeSO_4_·7H_2_O, 73.2; NaCl, 40.0; MgSO_4_·7H_2_O, 284.0; MnSO_4_·H_2_O, 6.50; KI, 0.68; Na_2_SeO_3_, 0.10; ZnSO_4_·7H_2_O, 131.93; Cellulose, 501.09.

### Cell lines and virus

Zebrafish embryonic fibroblast (ZF4) cells were purchased from American Type Culture Collection (ATCC number CRL-2050). Epithelioma papulosum cyprini (EPC) cells and SVCV (ATCC: VR-1390) were gifts by professor Jun-Fa Yuan (Huazhong agricultural university, Wuhan, Hubei, China). The SVCV was propagated in EPC cells at 28°C. The virus production was conducted according to previously described protocols (30). ZF4 and EPC cells were cultured at 28 °C in a 5% CO_2_ incubator in DMEM/F-12 medium (Corning, USA) and MEM medium (Corning, USA) with 10% fetal bovine serum (Gibco, Australia), respectively.

### SVCV challenge

SVCV challenge was conducted by the previous description (30). Briefly, fish were transferred to the incubator at 22°C to adapt before the SVCV challenge. The concentration of SVCV bath immersion was 10^6^ TCID_50_/ml. The death of zebrafish was recorded, and the survival rate was calculated for 14 days. On the 4^th^ day after the challenge, zebrafish were anesthetized with MS222, then the spleen was sampled and RNA was extracted, and the expression of antiviral genes of zebrafish was detected. The primer sequences are listed in **Table 2**.

**Table 2:** Primer sequences for *q*PCR.

Gene	Forward primer (5’–3’)	Reverse primer (5’–3’)
*rps11*	ACAGAAATGCCCCTTCACTG	GCCTCTTCTCAAAACGGTTG
*ifn-φ 1*	GAGCACATGAACTCGGTGAA	TGCGTATCTTGCCACACATT
*ifn-φ 2*	CCTCTTTGCCAACGACAGTT	CGGTTCCTTGAGCTCTCATC
*ifn-φ 3*	TTCTGCTTTGTGCAGGTTTG	GGTATAGAAACGCGGTCGTC
*mxb*	AATGGTGATCCGCTATCTGC	TCTGGCGGCTCAGTAAGTTT
*mxc*	GAGGCTTCACTTGGCAACTC	TTGTTCCAATAAGGCCAAGC
*rig-I*	TTGAGGAGCTGCATGAACAC	CCGCTTGAATCTCCTCAGAC
*irf3*	CAAAACCGCTGTTCGTGCC	CATCGTCGCTGTTGGAGTCCT
*irf7*	AGGCAGTTCAACGTCAGCTACCAT	TTCCACCAAGTTGAGCAATTCCAG
*tlr7*	GGGAGTTTCAGGACAGCCTT	TTCCTTGGCCACTCCAAAACT
*SVCV N*	TGAGGTGAGTGCTGAGGATG	CCATCAGCAAAGTCCGGTAT
*SVCV G*	TGCTGTGTTGCTTGCACTTATYT	TCAAACKAARGACCGCATTTCG
*il1b*	GGCTGTGTGTTTGGGAATCT	TGATAAACCAACCGGGACA
*il10*	ATAGGATGTTGCTGGGTTGG	GTGGATGAAGTCCATTTGTGC
*tnfα*	GCGCTTTTCTGAATCCTACG	TGCCCAGTCTGTCTCCTTCT
*mavs*	GTTCCCGGTCCAAGACACTA	TTGTCGCCTGAGTTGTTCTG

*rps11*, ribosomal protein S11; ifn-φ1, interferon-phi 1; *ifn-φ2*, interferon-phi 2; *ifn-φ3*, interferon-phi 3; *mxb*, myxovirus resistance B; *mxc*, myxovirus resistance C; *rig-I*, retinoic acid-inducible gene I; *irf3*, interferon regulatory factor 3; *irf7*, interferon regulatory factor 7; *tlr7*, toll-like receptor 7; *il1b*, interleukin 1 beta; *il10*, interleukin 10; *tnfα*, tumor necrosis factor α; *mavs*, mitochondrial antiviral signaling protein.

### Construction of SVCV standard plasmid and detection of whole fish viral load

The SVCV target fragment of PCR amplified was recovered by MiniBEST Agarose Gel DNA Extraction Kit (Takara). The amplified target fragment was inserted into the pLB vector (Tiangen) to construct the pLB-SVCV-FR plasmid, which was used as a standard plasmid to construct a calibration curve for estimating the copies of SVCV. Multiplex real-time quantitative RT-PCR (mqRT-PCR) was used to detect the viral load of whole fish. The concentration of pLB-SVCV-FR plasmid copies was adjusted to 10^10^ copies/μL. Then 10-10^9^ pLB-SVCV-FR plasmid copies were obtained by continuous dilution. The primer sequence of SVCV G protein was shown in **Table 2**. The sequence of SVCV G protein P primer is as follows: 5′-FAM-ATGAAGARGAGTAAACKGCCTGCAACAGA-TAMRA-3′. RNA was extracted from the whole zebrafish after the challenge. The cDNA synthesis was performed with the Fast-King gDNA Dispelling RT SuperMix (TIANGEN, KR118) using 1 μg of RNA and following the manufacturer’s indications. The standard plasmid was used to establish the standard curve of virus concentration. The PCR conditions were as follows: 95°C for 2 min, 40 cycles (95°C 3 s and 55°C 30 s). The standard curve was drawn with the CT value and the standard plasmid, and then the viral load was calculated according to the CT value obtained by different groups of whole fish cDNA.

### Detection of SVCV resistance of GCC-3 EPS in ZF4 cells

ZF4 cells with a concentration of 10^5^/well were inoculated into a 12-well plate and cultured at 28°C. GCC-3 EPS at the doses of 5, 10, and 15 μg/ml was added to the cell culture and incubated for 12 h. Subsequently, cells were infected with SVCV (0.1 MOI) for 24 h. Afterwards, the supernatant was collected to evaluate the TCID_50_ of SVCV as described previously (30). The total RNA of cells was extracted, and *q*PCR was used to determine the expression of the N protein of SVCV. The primer sequence of SVCV N is listed in **Table 2**.

### TBK1 gene silencing with small interfering RNA

In a 12-well plate, ZF4 cells in the logarithmic growth phase were inoculated. The reagent Lipofectamine RNAiMAX Transfection (Invitrogen) was used to transfect scrambled small interfering RNA (siRNA; negative control) and TBK1 siRNA into the plate according to the kit procedure. The sequence of TBK1 siRNA is as follows: Sense: GCGACAUCUUACACCGCAUTT; Antisense: AUGCGGUGUAAGAUGUCGCTT. After 24 h, 5 μg/ml GCC-3 EPS was added. SVCV at a concentration of 0.1MOI was administered after 6 h. *q*PCR was used to determine knocking down the efficacy of the siRNA therapy as well as the expression of the SVCV N protein.

### 16S *r*RNA gene sequencing

After a 2-week feeding, the intestinal contents of zebrafish were collected from each treatment group 4 h after the last feeding. Samples from six fish per tank were pooled as a replicate. DNA extraction, sequencing, and data analysis were conducted as previously described (26). Microbiota sequencing data in this study are available from the National Center for Biotechnology Information (NCBI) under accession number PRJNA841809.

### Culture of gut microbiota *in vitro*

The zebrafish were anesthetized with MS222 6 h after the last feeding. The intestinal contents of 5 fish per group were collected, pooled, and incubated in BHI liquid medium (Haibo, China) in an anaerobic incubator (Electrotek, England) containing 7% oxygen for 24 h and 48 h. The culture supernatants of the microbiota from control and EPS-fed zebrafish were collected by centrifugation and were then filtered through a 0.22 μm sterile tube top filter (Corning Inc., Corning). 30 μL supernatant was added to the ZF4 cell culture system and incubated for 24 h. Then cells were infected with SVCV (0.1 MOI) for 24 h. Afterwards, the cells were collected, and RNA extracted. The expression of the N protein of SVCV was quantified by *q*PCR to evaluate virus replication.

### Data analysis

Data were analyzed by GraphPad Prism 8.0. The Student’s *t*-test was used to compare the differences between the two groups. The survival rate after the SVCV challenge was analyzed by Log-rank (Mantel-Cox). Data were presented as mean ± SEMs. When *P* values were less than 0.05, the difference was considered significant.

## Results

### Effects of GCC-3 EPS on the antiviral ability of zebrafish

**Figure 1:**
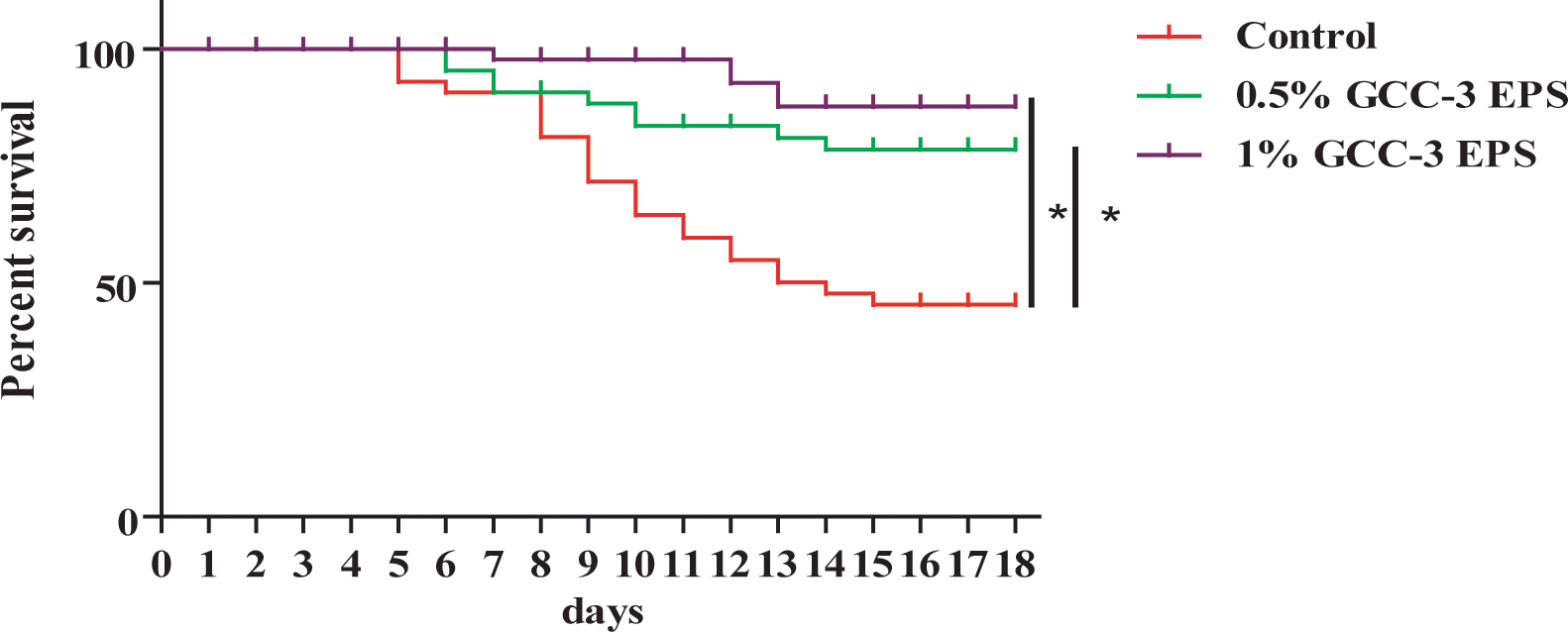
Effects of GCC-3 EPS supplementation on survival rate after SVCV infection. ^*^, *P* < 0.05 compared to the control group. Log-rank (Mantel-Cox) test for survival rate.

As shown in **Figure 1**, compared with the control group, dietary supplementation of 0.5% and 1% GCC-3 EPS significantly improved the survival rate of zebrafish post SVCV challenge after a 14-day feeding (**Figure 1**, *P* < 0.05). On the 5^th^ and 6^th^ days after the SVCV challenge, the viral load of the whole fish was detected. It was found that the load of SVCV in the whole fish of 0.5% and 1% GCC-3 EPS groups was significantly decreased (**Supplemental Figure 1**, *P* < 0.05).

### Effects of GCC-3 EPS on the antiviral immune response of zebrafish

**Figure 2:**
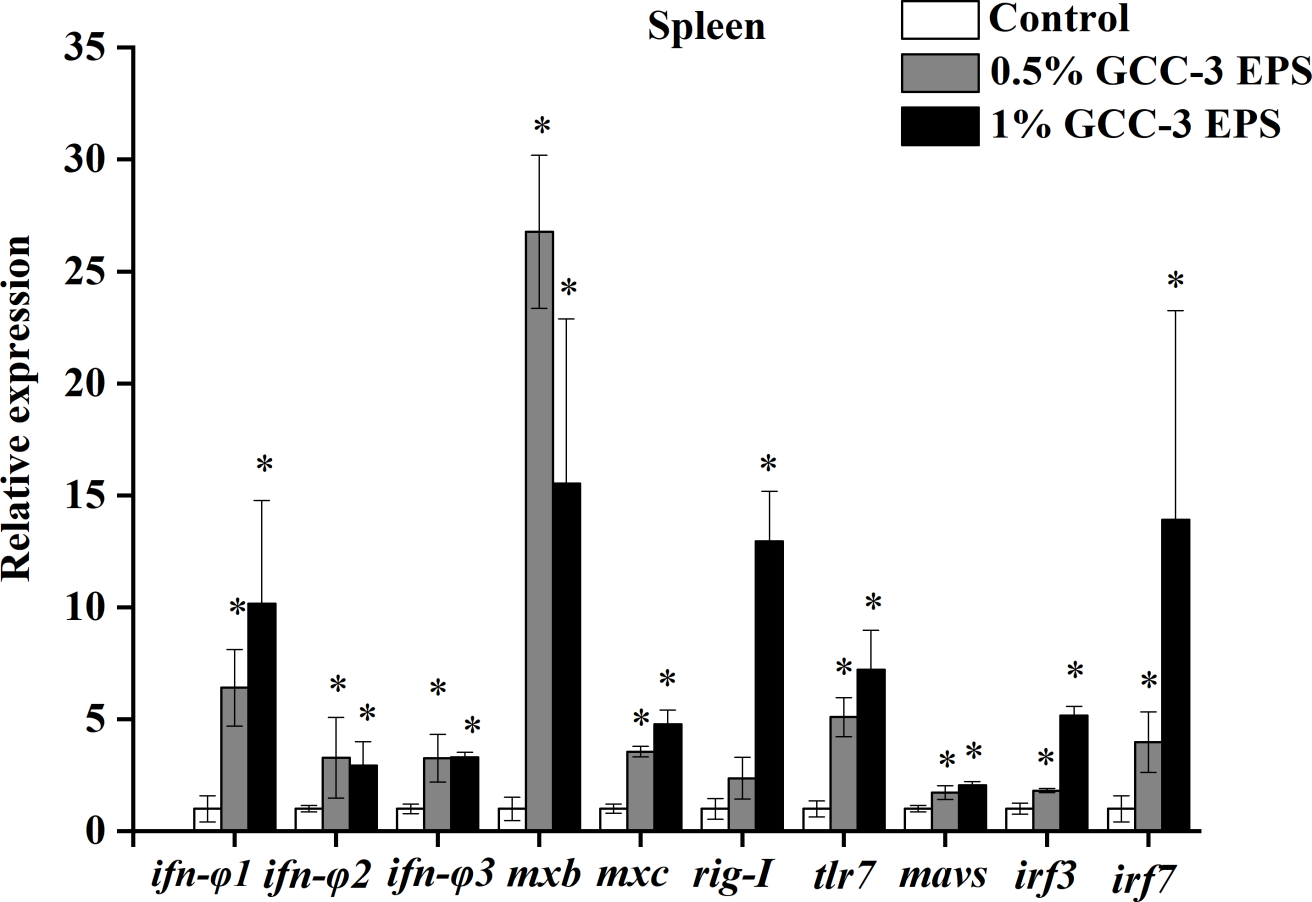
Relative mRNA expression of genes related to antiviral immunity in the spleen of zebrafish fed GCC-3 EPS and control diets after SVCV challenge. Data were expressed as the means ± SEMs (n = 6 biological replicates). ^*^, *P* < 0.05 compared to the control group. (Student’s t test).

As shown in **Figure 2**, dietary 0.5% and 1% GCC-3 EPS significantly up-regulated the expression of type I IFN genes in the spleen including *ifn-φ 1*、*ifn-φ 2*、*ifn-φ 3* and the expression of IFN-stimulated genes including *mxb* and *mxc* (*P* < 0.05). In addition, 0.5% and 1% GCC-3 EPS supplementation significantly increased the expression of virus recognition receptors related genes including *tlr7* and *rig-I* as well as the expression of downstream genes of IFN pathway such as *mavs*, *irf3* and *irf7* (**Figure 2**, *P* < 0.05).

### Effects of GCC-3 EPS on antiviral ability in ZF4 cells

**Figure 3:**
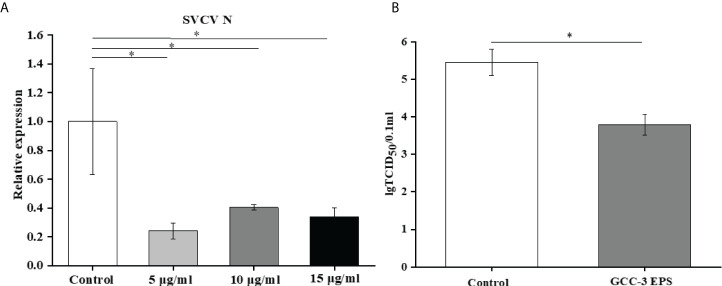
GCC-3 EPS suppressed SVCV replication in cells **(A)** and viral titer in supernatant of infected zebrafish embryonic fibroblast cells **(B)**. Data were expressed as the means ± SEMs (n = 5~6 biological replicates). ^*^, *P* < 0.05 compared to the control group.

GCC-3 EPS significantly reduced the expression of SVCV N protein at the three doses, indicating an inhibitory effect on viral replication (**Figure 3A**, *P* < 0.05). The expression of SVCV N protein in the 5 μg/mL GCC-3 EPS group was the lowest and therefore was selected for subsequent experiments. Consistent with the *q*PCR results, 5 μg/mL GCC-3 EPS significantly reduced SVCV titer in the supernatant of infected ZF4 cells (**Figure 3B**, *P* < 0.05).

### The antiviral function of GCC-3 EPS depends on the type I interferon signaling pathway

**Figure 4:**
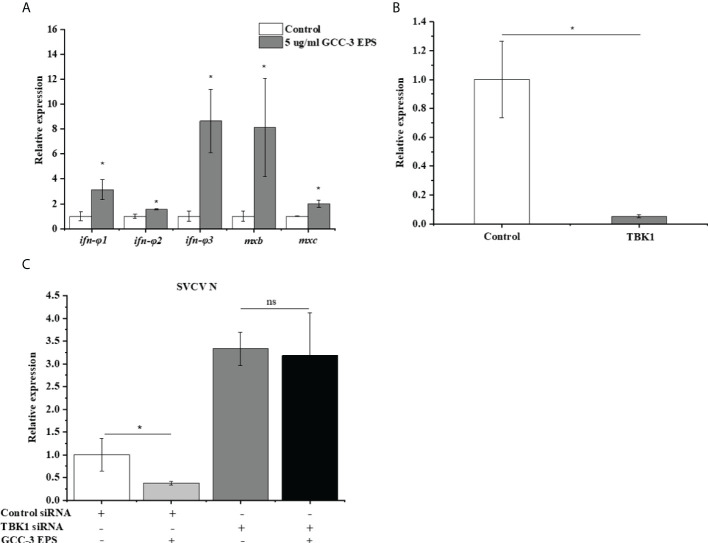
GCC-3 EPS activated the expression of genes related to type I interferon signaling pathway in zebrafish embryonic fibroblast cells **(A)**. Effect of small interfering RNA (siRNA) targeting TANK-binding kinase 1 (TBK1) on the expression of TBK1 **(B)**. Knocking down TBK1 abrogated the antiviral effect of GCC-3 EPS **(C)** in zebrafish embryonic fibroblast cells. Data were expressed as the means ± SEMs (n = 5~6 biological replicates). ^*^, *P* < 0.05 compared to the control group.

To eliminate the confounding effect of viral replication on antiviral gene expression, viral RNA mimics polyriboinosinic polyribocytidylic acid (poly (I: C)) (10 μg/ml) was transfected into ZF4 cells after GCC-3 EPS treatment, and the expression of genes related to the type I IFN signaling pathway was detected. Results showed that 5 μg/mL GCC-3 EPS treatment significantly increased the expression of *ifn-φ 1、ifn-φ 2、ifn-φ 3、mxb* and *mxc* after poly (I:C) stimulation compared with control, supporting that GCC-3 EPS can stimulate the type I IFN antiviral immunity (**Figure 4A**, *P* < 0.05). Therefore, GCC-3 EPS might exert antiviral effect by activating the IFN signaling pathway. To verify this hypothesis, siRNA of TBK1 was used to knock down the TBK1 gene in ZF4 cells (**Figure 4B**). After SVCV infection, the expression of SVCV N protein was detected to evaluate SVCV replication. Knocking down TBK1 significantly enhanced the expression of SVCV N protein in ZF4 cells versus control siRNA counterpart groups (**Figure 4C**), indicating the importance of IFN signaling pathway in suppressing SVCV infection. Interestingly, we found that after knocking down TBK1, there was no significant difference in SVCV replication between GCC-3 EPS group and control (**Figure 4C**, *P* > 0.05), supporting that the antiviral effect of GCC-3 EPS was mediated by type I IFN signaling pathway.

### Effects of GCC-3 EPS on the gut microbiota of zebrafish

**Figure 5:**
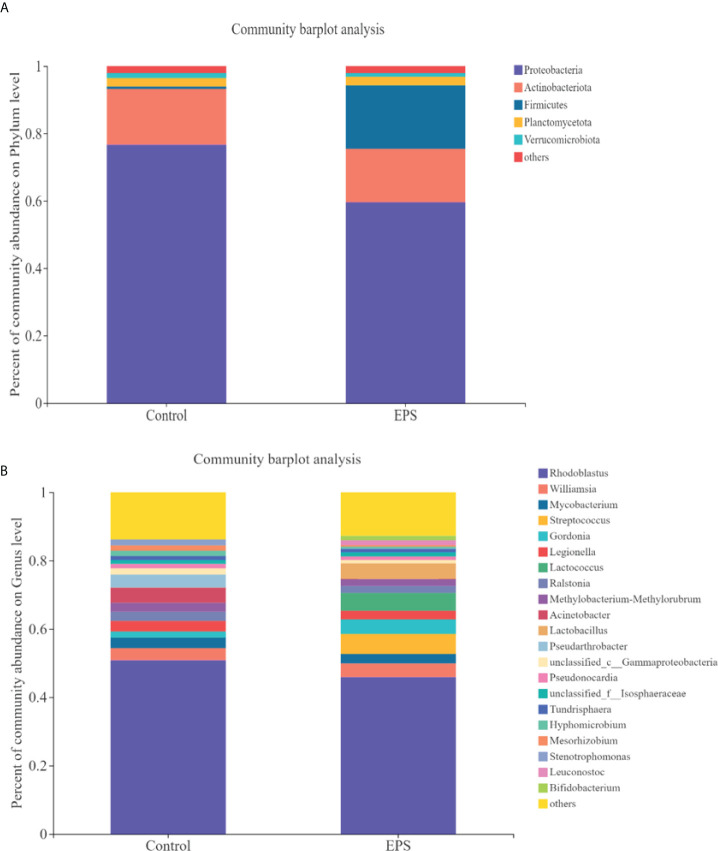
Effects of GCC-3 EPS on the composition of gut microbiota of zebrafish. Staked bar chart of relative abundance of the intestinal microbiota at the phylum level **(A)** and genus level **(B)**. Data were expressed as the means ± SEMs (n = 6 biological replicates). EPS represents the group of 1% GCC-3 EPS.

**Figure 6:**
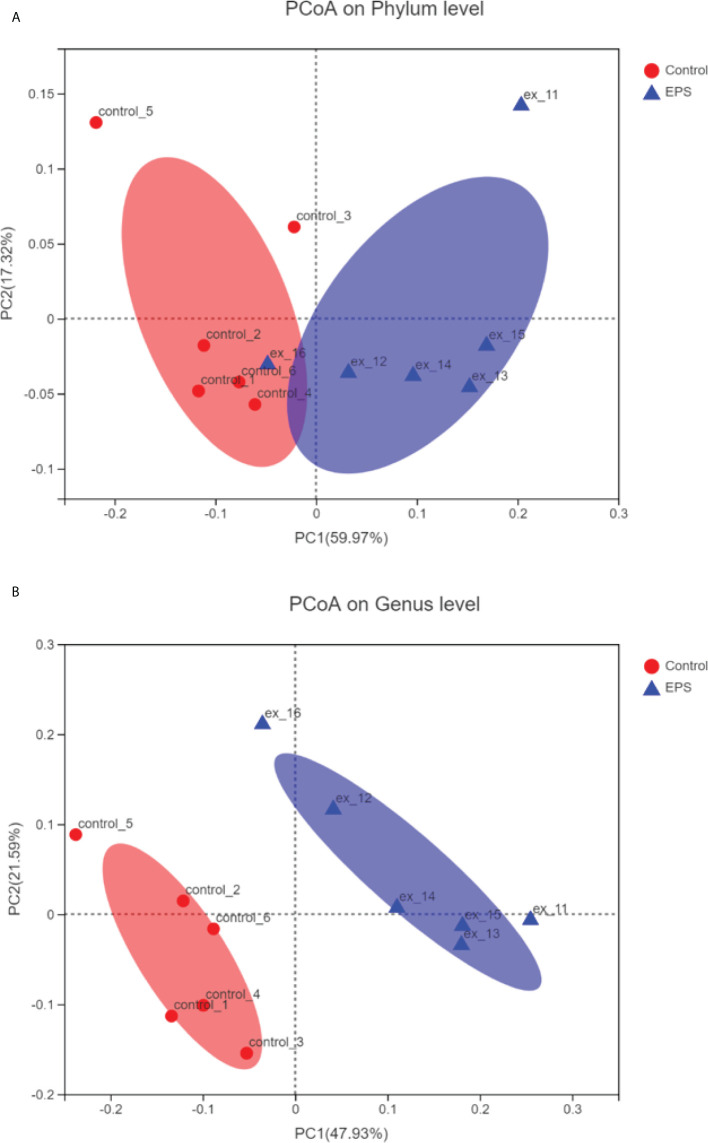
Principal coordinate analysis (PCoA) of control- and GCC-3 EPS-associated microbiota at the phylum level **(A)** and genus level **(B)**. Data were expressed as the means ± SEMs (n = 6 biological replicates). EPS represents the group of 1% GCC-3 EPS.

Compared to the control group, dietary GCC-3 EPS decreased the relative abundance of Proteobacteria and Actinobacteria while increased Firmicutes abundance at the phylum level (**Figure 5A**). At the genus level, the addition of GCC-3 EPS increased the abundance of *Lactobacillus* and *Bifidobacterium* (**Figure 5B**). Principal coordinate analysis (PCoA) analysis showed that there was a significant difference in the composition of gut microbiota between the control group and the GCC-3 EPS group at the phylum and genus level (**Figure 6**).

### Effects of culture supernatant of GCC-3 EPS-associated microbiota on SVCV replication

**Figure 7:**
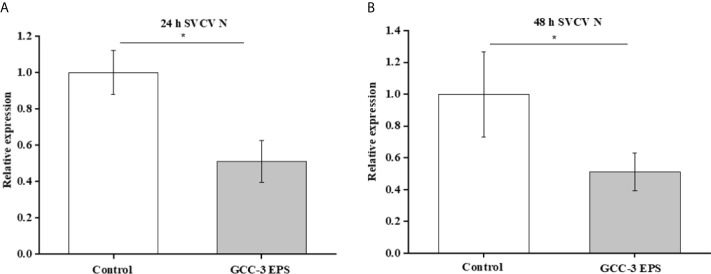
*In vitro* culture supernatant of gut microbiota of 1% GCC-3 EPS fed zebrafish suppressed SVCV replication in zebrafish embryonic fibroblast cells versus control microbiota counterpart. **(A)** 24 h culture supernatant, **(B)** 48 h culture supernatant. Data were expressed as the means ± SEMs (n = 6 biological replicates). ^*^, *P* < 0.05 compared to the control group.

The GCC-3 EPS-microbiota and control microbiota was suspended in BHI medium and were cultured in an anaerobic incubator containing 7% oxygen for 24 h and 48 h. The culture supernatants were added to ZF4 cells and the expression of SVCV N protein was detected after SVCV addition for 24 h. Results showed that the culture supernatant of GCC-3 EPS-associated microbiota significantly inhibited the expression of SVCV N protein of SVCV in ZF4 cells compared with the counterpart of control microbiota, suggesting that the GCC-3 EPS microbiota contributed to the antiviral effect (**Figure 7**, *P* < 0.05).

## Discussion

Dietary *Lactobacillus* EPS is known to have beneficial effects on the function of immune regulation (25–29). However, the effect of *Lactobacillus*-derived EPS on the antiviral ability of fish has not been reported. In the present work, 0.5% and 1% GCC-3 EPS were added to the diet of zebrafish, followed by SVCV challenge after a 14-day feeding. Results showed dietary 0.5% and 1% GCC-3 EPS improved the survival rate of zebrafish. Spleen is an important immune organ of fish (31). SVCV has been reported to attack the spleen after entering fish (2). Therefore, the spleen was collected to detect the expression of antiviral genes. We found that dietary 0.5% and 1% GCC-3 EPS increased the expression of type I IFN genes and IFN-stimulated genes in the spleen. IFN system is critical in combating viruses and can control the majority of viral infections in the absence of adaptive immunity (32, 33). Furthermore, dietary 0.5% and 1% GCC-3 EPS also up-regulated the expression of virus recognition receptors related genes including *tlr7* and *rig-I* and downstream genes of virus recognition receptors activation including *mavs*, *irf3* and *irf7* in the spleen. *rig-I* and *tlr7* are important cytoplasmic pattern-recognition receptors that stimulate the production of type I IFN in host innate immunity (34, 35). After activation, they can activate the expression of downstream genes including *mavs*, *irf3* and *irf7* (36). Taken together, these results showed that GCC-3 EPS enhanced the antiviral immune response of zebrafish, and the type I interferon signaling pathway might play an important role.

To further investigate the underlying mechanisms of the antiviral effect of GCC-3 EPS, we conducted experiments in ZF4 cells. Consistent with the *in vivo* results, we found that the addition of GCC-3 EPS reduced the expression of SVCV N protein, and the dose of 5 μg/mL exhibited the best effect. Poly(I:C) has been shown to be the most effective inducer of type I IFN (37, 38). The present results further demonstrated that GCC-3 EPS induced higher expression of type I IFN signaling pathway related genes after poly (I:C) stimulation versus control, indicating that GCC-3 EPS can activate the type I IFN signaling pathway at the cellular level. TANK-binding kinase 1 (TBK1) is a critical protein kinase in the synthesis of type I IFN and is important in antiviral innate immune response (39, 40). TBK1 can be activated by the mitochondrial antiviral signaling protein (*mavs*) signaling pathway mediated by the retinoic acid-inducible gene-I (*rig-I*) receptor in the cytoplasm (41). The activation of TBK1 affects the phosphorylation of IFN regulatory factor 3 and 7 (IRF3 and IRF7), which can induce the expression of type I IFN (42, 43). Notably, knockdown of the TBK1 gene blocked the effect of GCC-3 EPS on antiviral ability, indicating that the type I IFN signaling pathway is essential for the inhibitory effect of GCC-3 EPS against SVCV infection (44).

EPS is used as the source of energy and nutrients available to the gut microbiota and its addition can alter the composition of gut microbiota (45). Studies have shown that gut microbiota plays an important role in virus infection (13, 17, 46). In the present study, dietary GCC-3 EPS improved the composition of gut microbiota of zebrafish by increasing Firmicutes abundance and decreasing the relative abundance of Proteobacteria and Actinobacteria. PCoA analysis showed that there was a big difference in the composition of gut microbiota between the control group and the GCC-3 EPS group. Previous studies indicated that some bacterial species belonging to Firmicutes can protect fish against viral infection (17, 23, 24), suggesting that the higher abundance of Firmicutes might lead to an enhanced antiviral effect of EPS-associated microbiota. To initially evaluate whether GCC-3 EPS-associated microbiota contributes to the antiviral effect, we added the culture supernatant of GCC-3 EPS-associated microbiota to ZF4 cells and found the replication of SVCV in ZF4 cells was reduced as compared with the control microbiota culture supernatant group. These findings suggest that GCC-3 EPS-associated microbiota might contribute to the prevention of SVCV infection. However, the associated effectors need further investigation.

In conclusion, the present work indicates that dietary GCC-3 EPS can improve the antiviral ability of zebrafish. Studies using ZF4 cells showed that the antiviral function of GCC-3 EPS depended on the type I interferon signaling pathway. Further results showed that GCC-3 EPS altered the microbiota, and the EPS-associated microbiota might contribute to the antiviral effect. As a whole, our results provide novel insights into the antiviral effect of dietary supplemented *Lactobacillus* EPS against SVCV in zebrafish.

## Data availability statement

The datasets presented in this study can be found in online repositories. The names of the repository/repositories and accession number(s) can be found below: https://www.ncbi.nlm.nih.gov/, PRJNA841809.

## Ethics statement

The animal study was reviewed and approved by the Institute of Feed Research of the Chinese Academy of Agricultural Sciences chaired by the China Council for Animal Care (No. 2018-AF-FRI-CAAS-001).

## Author contributions

MX: wrote the paper and conducted the research; YL: conducted the research, formal analysis, and data curation; RO: resources and methodology; ER: resources and methodology; YY: analyzed the data; ZZ: analyzed the data; CR: research design, data curation, funding acquisition, and revising the draft; ZGZ: research design, data curation, funding acquisition, and revising the draft. All authors contributed to the article and approved the submitted version.

## Funding

This study was funded by National Key R&D Program of China (2018YFD0900400) and National Natural Science Foundation of China (31925038, 32122088).

## Conflict of interest

The authors declare that the research was conducted in the absence of any commercial or financial relationships that could be construed as a potential conflict of interest.

## Publisher’s note

All claims expressed in this article are solely those of the authors and do not necessarily represent those of their affiliated organizations, or those of the publisher, the editors and the reviewers. Any product that may be evaluated in this article, or claim that may be made by its manufacturer, is not guaranteed or endorsed by the publisher.
