# Supplementary material for: Dietary supplementation of exopolysaccharides from Lactobacillus rhamnosus GCC-3 improved the resistance of zebrafish against spring viremia of carp virus infection
Source: Front Immunol. 2022 Aug 5;13:968348. doi: 10.3389/fimmu.2022.968348 (PMC9389081; doi:10.3389/fimmu.2022.968348)
Supplement: Supplementary file 1 [file Image_1.pdf]

Dietary supplementation of exopolysaccharides from *Lactobacillus rhamnosus* GCC-3 improved the resistance of zebrafish against spring viremia of carp virus infection

Supplementary Material

Supplemental Figure

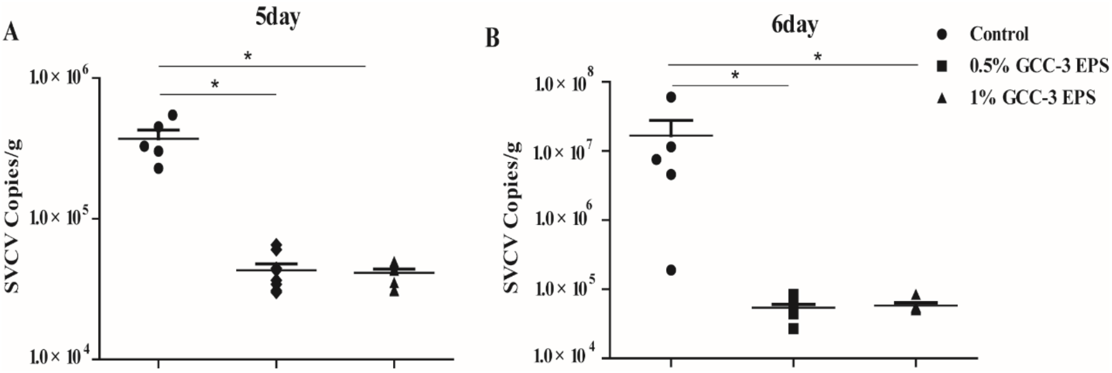

Supplemental Figure 1. Effects of GCC-3 EPS on viral load of zebrafish on the 5th and 6th days after SVCV infection (A and B). *$P < 0.05$.
